# Sigmoid Resection vs Conservative Treatment After Diverticulitis: Prespecified 4-Year Analysis of the LASER Randomized Clinical Trial

**DOI:** 10.1001/jamasurg.2025.0572

**Published:** 2025-04-09

**Authors:** Alexandre Santos, Panu Mentula, Tarja Pinta, Shamel Ismail, Tero Rautio, Risto Juusela, Aleksi Lähdesmäki, Tom Scheinin, Ville Sallinen

**Affiliations:** 1 Gastroenterological Surgery, University of Helsinki and Helsinki University Hospital, Helsinki, Finland; 2 Department of Surgery, Seinäjoki Central Hospital, Seinäjoki, Finland; 3 Department of Surgery, Medical Research Center, Oulu University Hospital, Oulu, Finland; 4 Department of Surgery, Vaasa Central Hospital, Vaasa, Finland; 5 Transplantation and Liver Surgery, University of Helsinki and Helsinki University Hospital, Helsinki, Finland

## Abstract

**Importance:**

Laparoscopic elective sigmoid resection is a treatment option for patients with recurring, persistent painful, or complicated diverticulitis, but outcomes of surgery compared with conservative treatment are unclear in long-term follow-up.

**Objective:**

To compare quality-of-life (QOL), complication, and recurrence outcomes of surgery vs conservative treatment in patients with recurring, persistent painful, or complicated diverticulitis.

**Design, Setting, and Participants:**

The open-label Laparoscopic Elective Sigmoid Resection Following Diverticulitis (LASER) randomized clinical trial was conducted in 6 Finnish hospitals. Ninety patients with recurring, persistent painful, or complicated diverticulitis were randomized (1:1) to elective sigmoid resection or conservative treatment from September 2014 to October 2018. Herein, outcomes are reported at 4-year follow-up using the intention-to-treat principle. Data analysis for this 4-year follow-up was performed from October 2023 to November 2024.

**Interventions:**

Laparoscopic elective sigmoid resection vs conservative treatment.

**Main Outcomes and Measures:**

Secondary outcomes, such as Gastrointestinal Quality of Life Index (GIQLI) scores, complications, and recurrences, within 4 years are reported using intention-to-treat and post hoc per-protocol analyses.

**Results:**

Of 90 enrolled patients (28 male [31%] with mean [SD] age of 54.11 [11.9] years; 62 female [69%] with mean [SD] age of 57.13 [7.6] years), 45 were randomized to elective sigmoid resection and 45 to conservative treatment. Among those randomized to conservative treatment, 14 of 44 (32%) underwent sigmoid resection within 4 years (patients with lower QOL on average). The mean (SD) GIQLI score was 115.3 (17.8) in the surgery group vs 109.8 (19.8) in the conservative treatment group (mean difference, 5.54 [95% CI, −2.98 to 14.06]) at 4 years. Recurrence of diverticulitis occurred in 6 of 38 patients (16%) (4 [10%] after surgery) in the surgery group vs 34 of 37 patients (92%) in the conservative treatment group. Severe postoperative complications occurred in 4 patients (10%) in the surgery group vs 5 patients (11%) in the conservative treatment group.

**Conclusions and Relevance:**

High crossover rates from conservative treatment to surgery indicate that patients with low QOL in the conservative treatment group often require surgical intervention; elective sigmoid resection did not improve QOL compared with conservative treatment in 4-year follow-up, even though it was effective in preventing recurrences of diverticulitis and did not lead to increased rates of postoperative complications. Upfront surgery may be preferable in patients with low QOL, but initial conservative treatment is an option for patients with near-normal QOL.

**Trial Registration:**

ClinicalTrials.gov Identifier: NCT02174926

## Key Points

**Question:**

Does sigmoid resection offer sustained improvement in quality of life compared with long-term outcomes of conservative treatment for patients with recurring, complicated, or persistent painful diverticulitis?

**Findings:**

In this randomized clinical trial comparing elective sigmoid resection vs conservative treatment with 4-year follow-up, one-third of patients initially randomized to conservative treatment underwent surgery eventually as they had persistent low quality of life; patients who were randomized to conservative treatment and did not undergo surgery had similar quality of life compared with patients randomized to upfront sigmoid resection. Early upfront surgery prevented recurrences effectively without increasing complications.

**Meaning:**

In patients with recurrent diverticulitis, surgery is a potential option to prevent recurrences and improve quality of life without increasing the risk of complications; however, patients with relatively normal quality of life may be equally satisfied with the conservative treatment option.

## Teaser

This prespecified 4-year analysis of the LASER randomized clinical trial compares quality-of-life, complication, and recurrence outcomes of surgery vs conservative treatment in patients with recurring, persistent painful, or complicated diverticulitis.

## Introduction

Diverticulosis is one of the most prevalent conditions in Western countries and seems to be increasing also in Eastern countries.^1,2,3^ Of patients having diverticulosis, approximately 4% have an episode of acute diverticulitis^4^ and one-fourth of these will have several recurrent episodes,^5,6^ making it a common condition to encounter in acute care. While the vast majority of acute diverticulitis cases are uncomplicated and resolve spontaneously,^6^ a portion of patients will have persistent pain even after the acute phase has passed.^7^

Elective sigmoid resection is a treatment option for patients with recurrent or persistent painful diverticulitis, as it improves quality of life (QOL) and prevents recurrent episodes of diverticulitis effectively, at least in the short term, over conservative treatment.^8,9,10,11^ High-quality evidence for long-term benefits and risks of surgery vs conservative treatment is scarce. This is reflected in the current guidelines, which recommend an individualized approach in patients with painful or recurring diverticulitis, with no clear criteria for surgery.^12,13,14,15,16^

To provide level 1 evidence regarding the benefits and risks of elective sigmoid resection vs conservative treatment for patients with recurrent, complicated, or persistent painful diverticulitis, we designed and conducted the multicenter Laparoscopic Elective Sigmoid Resection Following Diverticulitis (LASER) randomized clinical trial.^9^ We previously reported the outcomes up to 2-year follow-up.^10^ The results indicated a QOL benefit and good control of recurrent episodes but a higher risk of postoperative complications in patients randomized to surgery. Herein, we report outcomes at 4-year follow-up of the LASER trial.

## Methods

### Study Design

The LASER trial was a multicenter, parallel-group, open-label, individually randomized clinical trial in which patients with recurrent, complicated, or persistent painful diverticulitis were randomly assigned to receive either elective laparoscopic sigmoid resection or conservative treatment. The trial was conducted at 2 university hospitals (Helsinki University Hospital, Oulu University Hospital) and 3 community (central) hospitals (Etelä-Pohjanmaa Central Hospital, Vaasa Central Hospital, and Hyvinkää Hospital) in Finland. The trial was approved by the ethical committee of the Helsinki University Hospital and by the institutional review board at each center. Patients provided written informed consent prior to randomization. Results of 6-, 12- and 24-month follow-ups were previously published,^9,10^ and the methods used in the trial, baseline characteristics, and patient and public involvement information were described in detail. Therefore, only key methods are summarized here. The full trial protocol can be found in Supplement 1. The Consolidated Standards of Reporting Trials (CONSORT) reporting guidelines were followed in reporting this trial. Patients were assessed for eligibility between September 29, 2014, and October 10, 2018. Data analysis for this 4-follow-up was performed from October 2023 to November 2024.

### Participants

The study included patients who met at least 1 of the following criteria: (1) 3 or more episodes of left colon diverticulitis within a 2-year period with at least 1 episode verified using computed tomography (CT); (2) 1 or more episodes of complicated left colonic diverticulitis treated conservatively; or (3) prolonged pain or disturbance in bowel habits over 3 months after an episode of CT-verified acute left colonic diverticulitis. The study excluded patients with multimorbidity that precluded elective surgery; contraindications to laparoscopy; colonic stricture; fistula (such as colocutaneous, colovaginal, or vesical); active malignancy; previous resection of the sigmoid colon or rectum; acute diverticulitis that had not resolved (as evidenced by elevated inflammatory markers or fever); colonoscopy, sigmoidoscopy, or virtual colonoscopy not performed within 2 years; age younger than 18 years or older than 75 years; pregnancy; or inability to complete a health survey.

### Randomization

Patients were randomly allocated (1:1) to either elective laparoscopic sigmoid resection or conservative treatment. The patients, recruiters, health care professionals, outcome assessors, and data analyzers were not blinded to the allocated intervention.

### Procedures

Patients who were allocated to conservative treatment were provided with standardized written materials concerning diverticulosis and constipation. These materials recommended enhancing fiber consumption in the diet and included a prescription for a fiber supplement. For the group of patients allocated to elective laparoscopic sigmoid resection, surgery was scheduled within 3 months of randomization. Following the surgery, the patients received the same standardized written materials as those in the conservative treatment group.

Patients assigned to the conservative treatment group were expected to continue this treatment for a minimum of 6 months from the date of randomization, except in cases where an urgent surgical intervention was necessary, such as in cases of fistula, stricture, or perforation. The protocol allowed patients in the conservative treatment group to choose to undergo elective sigmoid resection after the 6-month period if they desired to do so. Also, patients had the option to withdraw consent to participate in the trial at any time.

### Outcomes

The LASER trial was conducted to assess the efficacy of treatment in patients with diverticulitis, with the primary outcome being the difference in Gastrointestinal Quality of Life Index (GIQLI) scores between randomization and 6 months after randomization. In our earlier reports, we presented these outcomes along with other measures assessable with the same time frame and up to 2 years.^9,10^ Secondary end points included GIQLI scores at 12, 24, 48, and 96 months, scores on the 36-Item Short Form Health Survey (SF-36) at 6, 12, 24, 48, and 96 months, recurrence and severity of recurrent diverticulitis (Hinchey classification), emergency surgery due to diverticulitis, elective sigmoid resection in patients who received conservative treatment, complications arising from elective sigmoid resection, mortality from any cause, complications of diverticular disease, and stoma rate. Herein, we present the results of the secondary outcomes at the 48-month follow-up, with the subsequent data to be reported separately on completion of the 8-year follow-up.

Patients were contacted by mail at 48 months. In case the patients did not respond to the letter or if the answers in the questionnaires were unclear, the patients were contacted by telephone. The data were collected prospectively using electronic case report forms.

### Statistical Analysis

According to sample size calculations for the primary outcome, the study aimed to recruit 133 patients. However, the trial was prematurely stopped due to significant differences in the primary outcome in the interim analysis.

The *t* test was used to compare continuous outcomes that were normally distributed (GIQLI, new cases of diverticulitis), with effect sizes reported as mean difference with 95% CI. The minimal clinically important difference for the GIQLI was estimated to range between 6.42 and 7.64.^17^ Continuous outcomes that were not normally distributed (Physical Component Summary and Mental Component Summary scores of the SF-36)^18^ at 48 months were compared using Mann-Whitney *U* test, and effect size is reported as *r* (*Z*/√N) without 95% CIs. Missing items in the GIQLI were imputed using multiple regression methods if the questionnaire was filled at least up to 75%. Isolated items were missing in 4 patients at 48 months. Except for GIQLI, cases with missing data were omitted from analyses and the number of missing data points are stated either in the text or tables. Categorical outcomes were compared using Fisher exact test (if expected cases in 1 cell were <5) or χ^2^ test, and effect size is reported as an odds ratio with 95% CI. The intention-to-treat principle was used for the main analysis, where patients were analyzed in the group to which they were randomized instead of the treatment they ultimately received. Given the high number of patients who eventually had surgery even though originally randomized to the conservative treatment group, a post hoc per-protocol analysis was also performed for secondary outcomes, where only patients who received the allocated treatment and did not cross over to the other group were included. We also performed a simple comparison of the differences in the GIQLI at baseline between the patients in the conservative treatment group who ultimately required surgery and those who did not. Two-tailed *P* < .05 was considered statistically significant. All analyses were performed using SPSS version 25 statistical software (IBM).

## Results

**Figure 1.**
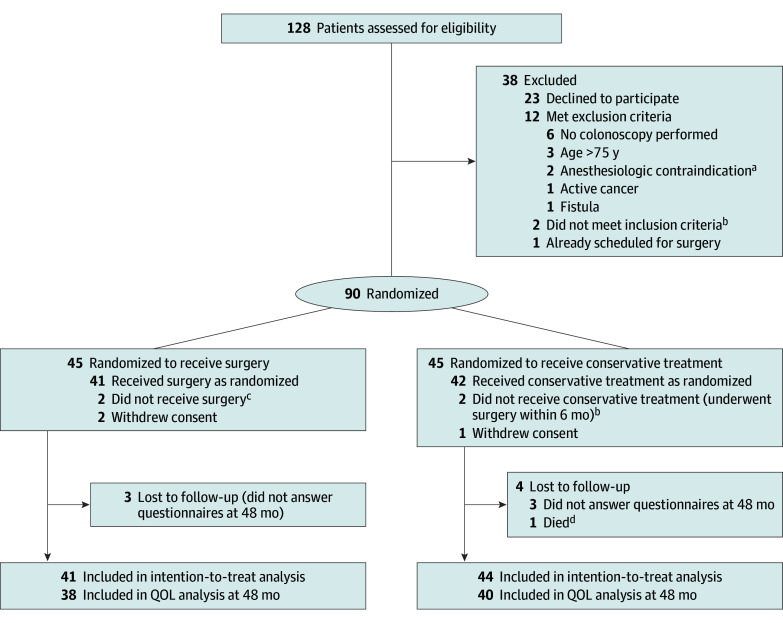
Flowchart of the Trial for the 48-Month Outcomes Excluded patients could meet more than 1 exclusion criteria. QOL indicates quality of life. ^a^Morbid obesity in 1 patient and myasthenia gravis in 1 patient. ^b^No computed tomography–confirmed diverticulitis. ^c^Did not meet inclusion criteria (right-sided diverticulitis). ^d^Death was unrelated to diverticular disease.

Between September 29, 2014, and October 10, 2018, a total of 128 patients were evaluated for eligibility. Of those, 90 patients were randomized and enrolled in the study (28 male [31%] with mean [SD] age of 54.11 [11.9] years; 62 female [69%] with mean [SD] age of 57.13 [7.6] years), with 45 receiving surgical intervention and 45 undergoing conservative treatment. Following exclusions, 85 patients were included in the intention-to-treat analysis, with 41 in the surgery group and 44 in the conservative treatment group (Figure 1). The baseline characteristics are provided in a previous article.^9^ The eTable in Supplement 2 shows baseline characteristics for the 78 patients with quality-of-life questionnaire responses available.

Among 44 patients initially randomized to conservative treatment, 12 (27%) underwent elective sigmoid resection within 48 months. The indication was recurrent diverticulitis and pain in 11 patients and chronic lower left abdominal pain in 1 patient. Notably, 1 of these patients also developed antibiotic allergies after successive antibiotic treatments. Two additional patients (5%) required emergency surgery due to a pericolic abscess that did not resolve with conservative treatment at the 11th and 16th month from randomization. In total, 14 of 44 patients (32%) randomized to conservative treatment underwent sigmoid resection within 48 months. Two of 41 patients (5%) randomized to surgery (included in the intention-to-treat analyses) declined to undergo sigmoid resection.

Eleven of 41 patients (27%) randomized to surgery had minor postoperative complications. Four patients (10%) had major (Clavien-Dindo grade III or higher) postoperative complications (Table 1).

**Table 1.** Operative Characteristics and Postoperative Complications at 48 Months’ Follow-Up

Characteristic	Patients, No. (%)	
	Surgery (n = 41)	Conservative treatment (n = 44)
Surgery		
Laparoscopy	38 (93)	12 (27)
Open^a^	1 (2)	2 (5)
Stoma in primary operation	0	1 (2)
30-d Postoperative complications by Clavien-Dindo grade		
I	9 (22)	1 (2)
Dermatitis	1 (2)	0
Pain	2 (5)	0
Seroma	1 (2)	0
Hematuria	1 (2)	0
Fever	1 (2)	0
Thrombophlebitis	1 (2)	0
Nausea	1 (2)	0
Superficial wound infection	1 (2)	1 (2)
II	2 (5)	0
Urinary tract infection	1 (2)	0
Anastomotic intraluminal bleeding	1 (2)	0
IIIa	2 (5)	1 (2)
Abscess, percutaneous drainage	2 (5)	0
Anastomotic intraluminal bleeding	0	1 (2)
IIIb	2 (5)	4 (9)
Anastomotic leakage, reoperation	2 (5)	3 (7)
Adhesions	0	1 (2)
IV	0	0
Late complication, incisional hernia within 48 mo	1 (2)	2 (5)

^a^ One patient in each group initially underwent laparoscopic surgery but it was converted to open surgery. One patient in the conservative treatment group underwent open surgery from the beginning of the procedure.

In the conservative treatment group, minor postoperative complications, such as superficial wound infection and intraluminal bleeding, were observed in 2 of 44 patients (5%), which resolved without intervention. Within 48 months from randomization, major postoperative complications occurred in 5 of 44 patients (11%), including 1 patient who had intraluminal bleeding that was treated endoscopically. Three patients had anastomotic leakage, which required emergency surgery. One patient in the surgery group and 2 in the conservative treatment group developed an incisional hernia within 48 months, and all underwent hernia repair. Temporary intended stoma was observed in 2 of 41 patients (5%) in the surgery group and 4 of 44 patients (9%) in the conservative treatment group (odds ratio, 1.95 [95% CI, 0.34-11.26]; *P* = .45); all 6 patients successfully underwent stoma reversal surgery within 48 months. No permanent stomas were done in either group. One patient from the conservative treatment group died, but the cause of death (trauma) was unrelated to diverticular disease.

Thirty-eight of 41 patients (93%) randomized to surgery and 40 of 44 patients (91%) randomized to conservative treatment responded to QOL questionnaires at the 48-month follow-up. In the intention-to-treat analyses, the mean GIQLI score at 48 months was higher in the surgery group compared with the conservative treatment group (mean [SD], 115.31 [17.86] vs 109.78 [19.79]; mean difference, 5.54 [95% CI, −2.98 to 14.06]; *P* = .38) (Table 2). No significant differences were noted in the Physical Component Summary and Mental Component Summary scores of the SF-36 between the groups at 48 months (Table 2). During the 48-month follow-up period, 34 patients in the conservative treatment group had recurrent diverticulitis, including 2 cases of complicated diverticulitis with abscess that required emergency surgery (Table 2). Within the same time frame, 6 patients in the surgery group had recurrent diverticulitis (Table 2). It is noteworthy that among these 6 patients, 1 developed diverticulitis after randomization but prior to undergoing sigmoidectomy and 1 patient declined surgery. At 48 months, patients in both groups were similarly satisfied with the treatment (Table 3). Although pain seemed more frequent and intense in the conservative treatment group compared with the surgery group, the observed difference did not reach statistical significance (Table 3).

**Table 2.** Outcomes Within 48 Months Analyzed Using Intention-to-Treat and Per-Protocol Principles

Outcome	Intention-to-treat analysis^a^				Per-protocol analysis			
	Surgery (n = 41)	Conservative treatment (n = 44)	*P* value	Effect size (95% CI)^b^	Surgery (n = 39)	Conservative treatment (n = 30)	*P* value	Effect size (95% CI)^b^
GIQLI score at 48 mo, mean (SD)^c^	115.31 (17.86)	109.78 (19.79)	.38	5.54 (−2.98 to 14.06)	116.25 (17.89)	111.38 (19.52)	.31	4.86 (−4.70 to 14.43)
SF-36 score at 48 mo, median (IQR)^d^								
PCS	50.55 (45.81-55.80)	47.27 (40.29-54.98)	.37	0.103^e^	50.55 (45.74-56.15)	48.16 (43.72-56.09)	.69	0.052^e^
MCS	55.53 (51.47-59.84)	53.85 (40.83-59.70)	.45	0.087^e^	55.37 (50.73-59.93)	56.21 (42.24-60.15)	.93	0.011^e^
Recurrent episodes of diverticulitis within 48 mo, No. (%)^f^								
Any	6 (16)	34 (92)	<.001	60.44 (13.93 to 262.25)	5 (14)	20 (87)	<.001	41.33 (8.88 to 192.38)
Hinchey classification								
0 and Ia	6 (16)	31 (84)	…	…	5 (14)	19 (83)	…	…
Ib	0	3 (8)	…	…	0	1 (4)	…	…
II	0	0	…	…	0	0	…	…
III	0	0	…	…	0	0	…	…
IV	0	0	…	…	0	0	…	…

Abbreviations: GIQLI, Gastrointestinal Quality of Life Index; MCS, Mental Component Summary; PCS, Physical Component Summary; SF-36, 36-Item Short Form Health Survey; ellipses, not calculated.

^a^ In the intention-to-treat cohort, 2 patients (5%) included in the surgery group had not undergone surgery and 14 patients (32%) included in the conservative treatment group had undergone surgery.

^b^ Effect size indicates the mean difference (95% CI) for continuous variables and odds ratio (95% CI) for categorical variables.

^c^ For both the intention-to-treat analysis and the per-protocol analysis, data were missing in 3 patients in the surgery group and 4 patients in the conservative treatment group.

^d^ For both the intention-to-treat analysis and the per-protocol analysis, data were missing in 5 patients in the surgery group and 4 patients in the conservative group.

^e^ Effect size is calculated as *r* = *Z*/√N without 95% CI.

^f^ For both the intention-to-treat analysis and the per-protocol analysis, data were missing in 3 patients in the surgery group and 7 patients in the conservative group. Percentages are given per valid patients (intention-to-treat analysis: n = 38 in the surgery group and n = 37 in the conservative treatment group; per-protocol analysis: n = 36 in the surgery group and n = 23 in the conservative treatment group).

**Table 3.** Patients’ Perceptions and Pain at 48 Months From Randomization Analyzed Using Intention-to-Treat and Per-Protocol Principles^a^

Outcome	Intention-to-treat analysis			Per-protocol analysis		
	Surgery	Conservative treatment	*P* value	Surgery	Conservative treatment	*P* value
Patient satisfaction with assigned treatment at 48 mo, No. (%)	(n = 38)	(n = 37)		(n = 36)	(n = 24)	
Satisfied	34 (89)	31 (84)	.73	33 (92)	21 (88)	.47
Not satisfied	1 (3)	1 (3)		1 (3)	0	
Could not tell	3(8)	5 (14)		2(6)	3 (13)	
Pain at 48 mo, No. (%)	(n = 39)	(n = 40)		(n = 37)	(n = 26)	
No pain	19 (49)	11 (28)	.05	19 (51)	6 (23)	.02
Once/mo	14 (36)	17 (43)		12 (32)	13 (50)	
Once/wk	3 (8)	5 (13)		3 (8)	4 (15)	
Few times/wk	2 (5)	4 (10)		2 (5)	1 (4)	
Every day	1 (3)	2 (5)		1 (3)	1 (4)	
Several times/d	0	1 (3)		0	1 (4)	
All the time	0	0		0	0	
VAS score for pain at 48 mo	(n = 38)	(n = 38)		(n = 36)	(n = 24)	
Mean (SD)	2.03 (2.11)	2.50 (1.98)	.68	1.81 (1.80)	2.88 (1.99)	.04

Abbreviation: VAS, visual analog scale.

^a^ In the intention-to-treat analyses, 14 patients (32%) included in the conservative treatment group had undergone surgery and 2 patients (5%) included in the surgery group had not undergone surgery. The sample sizes shown for each outcome indicate the number of patients who responded to the questionnaire at 48 months’ follow-up.

**Figure 2.**
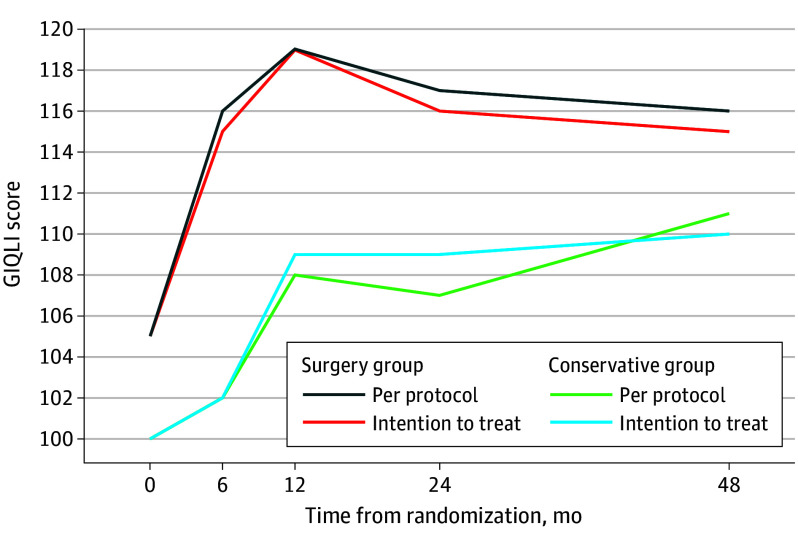
Gastrointestinal Quality of Life Index (GIQLI) Scores at Baseline and Follow-Up in Patients Randomized to Either Conservative Treatment or Surgery

For the post hoc per-protocol analysis, a total of 69 patients were included. Of these, 39 patients were randomized to the surgical group and underwent surgery, while the remaining 30 patients were randomized to conservative treatment and did not undergo sigmoidectomy within the 48-month study period. At 48 months, no significant differences were observed between the groups for QOL outcomes (GIQLI, SF-36), but recurrences occurred significantly more often in the conservative treatment group than in the surgery group (20 of 23 patients [87%] vs 5 of 36 patients [14%]; *P* < .001) (Table 2). Although patients were similarly satisfied with the treatment in both groups, pain frequency and intensity were higher in the conservative group (Table 3). Figure 2 illustrates the GIQLI in both groups during 4-year follow-up in both intention-to-treat and per-protocol cohorts.

Baseline QOL of patients randomized to conservative treatment but undergoing sigmoid resection in the follow-up was analyzed post hoc. Patients who were randomized to conservative treatment but underwent surgery within 48 months (n = 14) had lower baseline scores compared with patients who stayed in conservative treatment (n = 30) (mean [SD] GIQLI score, 104.58 [19.60] vs 90.79 [20.80]; mean difference, 13.79 [95% CI, 0.36-27.11]; *P* = .04). The surgery improved GIQLI scores on average by 17.57 GIQLI points in patients crossing over to surgery after initial conservative treatment (n = 14; mean [SD] GIQLI score, 91.25 [25.17] at the latest time point before crossing over to surgery vs 108.82 [15.75] at the first time point after surgery; mean difference, 17.57 [95% CI, 6.99-28.15]; *P* = .004).

## Discussion

In this prespecified 4-year follow-up analysis of the LASER trial, no significant differences were noted in QOL between patients with recurrent, complicated, or persistent painful diverticulitis randomized to elective sigmoid resection vs conservative treatment in either intention-to-treat or per-protocol analyses. However, surgery was highly effective in preventing recurrent episodes of diverticulitis, as only 10% undergoing upfront surgery had a recurrent episode after surgery compared with almost 90% of patients in the conservative treatment group. While 95% of patients randomized to surgery underwent sigmoid resection, approximately one-third of patients randomized to conservative treatment either opted for elective surgery or needed emergency surgery within 4 years from randomization. It is of note that even as two-thirds of patients in the conservative treatment group did not undergo surgery, the rate of major complications (Clavien-Dindo grade III or higher) was similar between the whole groups. This is reassuring in a sense that early upfront surgery did not increase risk. On the contrary, this indicates that patients who postpone surgery would likely have a higher risk of major postoperative complications should they need surgery later. The rate of major complications in the surgery group was 10% (4 of 39 patients undergoing surgery) compared with 36% (5 of 14 patients undergoing surgery) in patients starting with conservative treatment but eventually undergoing surgery. Of note, the stoma rate (2 in the surgery group vs 4 in the conservative treatment group) and the incisional hernia rate (1 in the surgery group vs 2 in the conservative treatment group) were double in patients randomized to conservative treatment, further favoring early surgery. These results are in contrast with the outcomes of the DIRECT trial,^11^ where complications occurred more often in patients randomized to surgery (any postoperative complication, 45% in the surgery group vs 13% in the conservative treatment group; major complication [Clavien-Dindo grade III or higher], 21% vs 2%, respectively). These differences between the trials might be due to different patient cohorts but also due to the fact that half of the patients randomized to the conservative treatment group undergoing surgery had their surgery within 6 months from inclusion in the DIRECT trial. As such, the surgery for these patients was not postponed, but instead could be considered to be early upfront surgery.

The lack of difference in QOL measurements at 4-year follow-up is in contrast with the results of the LASER trial at earlier time points (6, 12, and 24 months), where higher QOL was noted in the surgery group,^9,10^ but also with the 5-year results of the DIRECT trial.^11^ We found a difference of 5 GIQLI points between the groups at 4 years, which is neither clinically meaningful nor statistically significant. The DIRECT trial reported a difference of 10 GIQLI points at 5 years, which is both a clinically meaningful and statistically significant difference, even though the crossover rate from the conservative treatment group to surgery was higher (46%) than in the LASER trial (32%). The reasons behind this discrepancy and the lack of difference in the LASER trial can be speculated. First, the baseline characteristics of the patients included in the trials differ. While most patients in the LASER trial had recurrent diverticulitis (78%), these patients accounted for only 17% in the DIRECT trial. On the other hand, most patients in the DIRECT trial had persistent pain (62%), but persistent pain was present in only 6% of patients in the LASER trial. These differences in the indications are reflected on the baseline QOL measurements; the baseline mean GIQLI score in the DIRECT trial was 10 points lower than in the LASER trial (92 vs 102, respectively). Further, the baseline mean GIQLI score was 14 points lower in patients who initially started conservative treatment but crossed over to surgery compared with the ones who continued to receive conservative treatment. Thus, it seems evident that patients who had difficult symptoms opted for surgery, and those with no or only mild symptoms continued conservative treatment, explaining the lack of difference between the groups in intention-to-treat analyses (staying in the analyses with improved QOL after crossing to surgery) and in per-protocol analyses (excluding the patients crossing over to surgery, leaving only patients with good QOL in the analyses) in the 4-year follow-up of the LASER trial.

In this study, we observed a higher recurrence rate of diverticulitis compared with the DIRECT trial (92% vs 30%, respectively).^11^ This disparity can be attributed to differences in baseline characteristics between the 2 studies, as a majority of patients in the LASER trial had a history of recurrent diverticulitis (78%) compared with only 17% in the DIRECT trial. This finding aligns with existing literature, which indicates that the risk of recurrence increases with the number of prior diverticulitis episodes.^6^

### Limitations

The LASER trial has several limitations that should be considered when interpreting the results. First, the trial was prematurely terminated, although this was done according to the prespecified criteria in the study protocol. Second, the sample size can be considered relatively small. The trial had been running for 4 years before termination, and trials comparing surgery vs conservative treatment are challenging. Similar difficulties with recruitment were observed in the DIRECT trial.^8^ Third, the study cohort includes patients with 3 different inclusion criteria and can be considered heterogeneous in this respect. Fourth, the trial did not incorporate sham surgery to blind the intervention, as this was deemed likely to further complicate participant recruitment. Consequently, the potential influence of a placebo effect on the QOL outcomes cannot be excluded. However, the LASER trial has several strengths. Being randomized, it lacks several shortcomings of retrospective or noncontrolled studies and gives a realistic view of the 2 different treatment options in the long-term follow-up. Also, the response rate to QOL questionnaires was very high (92%) at 4 years. The follow-up of the patients is still continuing, and we will report outcomes at 8 years as they become assessable for all patients in 2026.

## Conclusions

In conclusion, several implications for clinical practice can be considered. First, elective sigmoid resection improved QOL over conservative treatment in patients with recurrent, complicated, or persistent painful diverticulitis. This improvement must be weighed against the risks of surgery, but patients can also be advised that the risk of complications is similar regardless of the initial choice between surgery and conservative treatment and is likely higher in individuals who start with conservative treatment but need surgery later. On the other hand, patients who have recurrent diverticulitis but no or only mild symptoms (good QOL scores) are likely to be satisfied with conservative treatment even if episodes of uncomplicated diverticulitis are highly likely to occur. Patients with 3 or more episodes of diverticulitis should be offered elective surgical options, and patients with lowered QOL are the ones especially benefiting from early surgery.

## Notes

Supplement 1. Trial Protocol

Supplement 2. **eTable.** Baseline Characteristic of Patients Included in Quality-of-Life Analyses

Supplement 3. Data Sharing Statement
