# Supplementary material for: Sigmoid Resection vs Conservative Treatment After Diverticulitis: Prespecified 4-Year Analysis of the LASER Randomized Clinical Trial
Source: JAMA Surg. 2025 Apr 9;160(6):615–22. doi: 10.1001/jamasurg.2025.0572 (PMC11983291; doi:10.1001/jamasurg.2025.0572)
Supplement: Supplement 1. — Trial Protocol [file jamasurg-e250572-s001.pdf]

LASER trial. LAparoscopic elective Sigmoid resection following divERticulitis - A multicenter, prospective, randomized clinical trial.

Finnish name: Laparoskooppinen elektiivinen sigmoidresektio divertikuloosin vuoksi - randomoitu prospektiivinen monikeskustutimus

Research plan v.1.6 (19.3.2014)

Alex Santos[1], Panu Mentula[1], Suvi Rasilainen[2], Tero Rautio[3], Arto Turunen[4], Samuli Aho[5], Tarja Pinta[6], Mikael Victorzon[7], Jukka Karvonen[8], Mirjami Uotila-Nieminen[9], Eero Kangas[10], Juha Rinne[11], Anna Fagerström[12], Tom Scheinin[1], Ville Sallinen[1]; LASER trial study group

LASER trial study group members: Matti Kairaluoma[5], Anu Erlich[5], Jan Andersén[7], Hillar Jääger[7], Heini Savolainen[12], Selja Koskensalo[1], Olli Kruuna[1], Heikki Huhtinen[8], Pirita Varpe[8]

[1] Helsinki University Central Hospital, Surgical Hospital

[2] Helsinki University Central Hospital, Jorvi Hospital

[3] Oulu University Hospital

[4] Kanta-Häme Central Hospital

[5] Central Finland Central Hospital

[6] South Ostrobothnia Central Hospital

[7] Vaasa Central Hospital

[8] Turku University Central Hospital

[9] North Karelia Central Hospital

[10] South Karelia Central Hospital

[11] Päijät-Häme Central Hospital

[12] Kuopio University Hospital

Background knowledge

Diverticulosis is an increasingly common ailment in the aging population. An estimated 25% develop acute diverticulitis (Biondo et al., 2012). The prognosis of acute diverticulitis is related to its severity measured according to the Hinchey classification. The mildest diverticulitis heals on its own, while mortality from diverticulitis that causes fecal peritonitis is high (Wasvary et al., 1999). Peritoneal inflammation caused diverticulitis usually leads to an emergency sigmoid resection and possible intestinal contents diversion through the stoma. In contrast, milder forms of acute diverticulitis are usually treated conservatively with an intravenous antibiotic and abscesses are drained.

It has previously been recommended that patients with recurrent diverticulitis undergo elective sigmoid resection to avoid further problems with complicated diverticulitis (Rafferty et al., 2006). However, retrospective work has found that the most difficult diverticulitis episode is usually the very first one, and the occurrence of complicated diverticulitis decreases with the recurrence of diverticulitis (Chapman et al., 2006; Ritz et al., 2011). Second, elective surgery for complicated diverticulitis (e.g., abscess) has been recommended (Rafferty et al., 2006). However, this recommendation has also recently been questioned (Gaertner et al., 2013). On the other hand, elective sigmoid resection has been shown to improve quality of life in selected patients with either recurrent diverticulitis or complicated diverticulitis (Forgione et al., 2009; Pasternak et al., 2011).

Current knowledge of who should perform elective sigmoid resection is very limited, is based on retrospective comparative series or prospective studies that lack a control group. To date, no randomized, prospective study comparing surgical treatment to conservative treatment in patients with a relative indication (recurrent diverticulitis, complicated diverticulitis, pain after acute diverticulitis) to elective sigmoid resection has been published. An extensive recent treatment recommendation for diverticulitis cannot provide definitive recommendations for the selection of patients for elective sigmoid resection due to the lack of randomized work (Andersen et al., 2012).

The main objective of the study is to investigate the effect of elective sigmoid resection on gastrointestinal quality of life by comparing surgical treatment to conservative treatment in a randomized prospective multicenter study. The secondary objective is to investigate recurrence, mortality, and surgical complications of diverticulitis.

Hypothesis

Elective sigmoid resection improves quality of life and reduces recurrence of diverticulitis in follow-up.

Procedures

The study sites

Helsinki University Central Hospital, Surgical Hospital

Helsinki University Central Hospital, Jorvi Hospital

Oulu University Hospital

Kanta-Häme Central Hospital

Central Finland Central Hospital

South Ostrobothnia Central Hospital

Vaasa Central Hospital

Turku University Central Hospital

North Karelia Central Hospital
South Karelia Central Hospital
Päijät-Häme Central Hospital
Kuopio University Hospital

Intervention Groups

1. Selective sigmoid resection

2. Conservative treatment (lifestyle guidelines, fiber supplement)

Study Patients

Inclusion criteria

1. Repeat left-side colon diverticulitis (at least 3, within a 2 years period, at least one CT confirmed)

2. Previously complicated diverticulitis * (excluding diverticulitis with only pericolar air)

3. CT-secured left. after-pain or gut side of diverticulitis of colon difficulties in the operation, which lasted for more than 3 months after acute treatment * Complicated diverticulitis refers to acute vascularization verified by computed tomography. side diverticulitis of colon, which involves fistula, stricture, or intestinal abscess found outside the computed aircraft.

Exclusion criteria

1. Multimorbidity that prevents elective shear consideration

2. Obstacle to laparoscopy (ex. anesthetic cause)

3. Colon stricture

4. Fistula (colocutan, colovesical, colovaginal, etc.)

5. Active cancer

6. Previous resection of the left colon or rectum

7. Acute diverticulitis that has not resolved (inflammatory values have returned to normal, fever has stopped)

8. Colonoscopy / sigmoideoscopy or colon computed tomography (virtual colonoscopy) has not been performed within 2 years of randomisation

9. Age <18 or> 75 years

10. Pregnancy

11. Inability to answer quality of life surveys (eg dementia, psychiatric illness, etc.)

Randomization

The patient is randomized 1: 1 for either elective sigmoid resection or conservative treatment. Patients are stratified randomly based on inclusion criteria so that there are equal numbers of patients from each inclusion group in both randomization groups. Randomization is done using a computer centrally from the HUCH. In practice, the person performing the randomisation connects to the HUCH server via the Internet, which provides the researcher with information about the patient's randomisation team. Randomization is performed in connection with an outpatient visit, after the patient has first given written consent to participate in the study. Based on the group, treatment is selected, i.e. the patient is either placed in a surgical queue for elective sigmoid resection or given lifestyle instructions as well as instructions on how to use fiber supplementation. Upon randomization, the patient completes SF-36 and GIQLI quality of life questionnaires. Patients are analyzed according to the intention-to-treat principle according to the original randomization group.

Intervention

1. Elective laparoscopic sigmoid resection

The patient is placed in a surgical queue for elective laparoscopic sigmoid resection. Surgery is performed within a maximum of 3 months of randomisation. CRF # 3 is filled just before surgery (e.g., during a preoperative visit). CRF # 2 is replenished as the patient returns home after elective sigmoid resection. After the operation, the patient is given lifestyle instructions as well as a recommendation for the use of a fiber supplement.

Technique of laparoscopy

1. Port setting free

2. Distal cleavage site in the upper rectum, below the promontory

3. Proximal cleavage site in oral sigmoid or descending colon

4. Flexura lienalis is mobilized as needed to obtain a non-tightening seam

5. The aim is to keep the blood vessels as good as possible. A. mesenterica inferior and a. Rectalis superior are sought to be spared. However, if necessary, the blood vessels can be severed, for example to provide a non-tight anastomosis or if the dissection conditions are difficult.

6. Intestinal anastomosis with ILS

2. Conservative treatment

Lifestyle guidelines in writing

- Duodecim Medical Publications Information to the patient: The intestinal diverticular disease, 12/11/2012 Pertti Black River (www.terveysportti.fi) (Look annexes)
- Medical book Duodecim Information for the patient: Constipation 8.10.2012 Pertti Mustajoki (www.terveysportti.fi) (look annexes)

Oral recommendation on the use of fiber supplement.

Action in the event of elective sigmoid resection in a patient randomized to conservative treatment:

Conservative treatment should be tried for at least 6 months of randomisation (= until the first quality of life survey) before it can be concluded that it has failed and the patient referred to elective sigmoid resection. Of course, surgical treatment can be performed earlier if the patient has developed absolute indications for elective sigmoid resection (eg fistula, stricture). If a patient randomized to conservative treatment has to be treated with elective sigmoid resection, the patient should complete the GIQLI and SF-36 quality of life questionnaires at the time of surgery. After surgery, the patient is followed according to the original follow-up schedule (i.e., surveys just before surgery (preoperative visit, etc.), 6 months, 12 months, 24 months, 48 months, and 96 months after the initial randomisation). In accordance with normal research ethical principles, the patient may also opt out of the study altogether, at which point his or her follow-up will cease and he or she will be treated according to normal treatment lines and at the discretion of the attending physician.

Variables to be examined

Primary outcome

Gastrointestinal Quality of Life Index (GIQLI) - difference between 0 months and 6 months from randomisation

Secondary variables

1. Gastrointestinal Quality of Life Index (GIQLI) at 12 months, 24 months, 48 months and 96 months of randomisation
2. SF-36 quality of life survey score at 6 months, 12 months, 24 months, 48 months and 96 months from randomisation
3. Recurrence of diverticulitis and Hinchey class
4. Undergoing emergency surgery due to diverticulosis / diverticulitis
5. Undergoing elective sigmoid resection (conservative treatment group)
6. Complications of elective sigmoid resection
7. Mortality (for any reason in both groups)
8. Complications of diverticulosis (fistula, stricture)
9. Stomach (permanent / temporary)

Tracking

The attached flow chart shows patient randomisation, timing of treatment procedures, and follow-up. Patients are assigned a playing time 6 months after randomisation to inquire about well-being and a decision can be made on a possible change in treatment (a patient in the conservative treatment group should be referred for elective sigmoid resection).

Patients are also monitored by mailed questionnaires (SF-36, GIQLI, and a questionnaire prepared by researchers, see appendix) just before surgery (preoperative visit, etc.) and 6 months, 12 months, 24 months, 48 months, and 96 months after randomisation. The same questionnaires are also completed just before elective sigmoid resection. If there is any ambiguity in the answers to the questionnaire, the researcher will, if necessary, contact the patient by telephone to clarify the ambiguities.

The researcher at each center collects a questionnaire from his or her own center and also checks the entries in the patient records in his or her own area. Based on these, the researcher fills out a CRF # 3 form, which is therefore completed at each follow-up time point.

Costs

The study is not expected to incur significant additional costs, as elective sigmoid resection is already a normal treatment and inclusion criteria follow general treatment recommendations. The only costs come from the phone call made to the patient (at 6 months) and the postage costs of the questionnaires. Research may even save hospital costs, as half of patients are randomized to conservative care and no surgical costs are involved.

Parameters to be collected

Case Report Form (see appendix). Case report form # 1 is completed at the time of randomization (outpatient visit). Case report from # 2 is completed electronically when the patient is discharged. Case report form # 3 is completed at each follow-up time point (just before surgery (preoperative visit, etc.), 6 months, 12 months, 24 months, 48 months, 96 months).

The sample size

In previous publications, preoperative GIQLI has been 95-100 (SD 22) and postoperative GIQLI 112-114 (SD 19-22) (Forgione et al., 2009; Pasternak et al., 2011). The sample size calculation was made using these values, and the study aims to show a difference of 12 GIQLI points, the standard deviation being assumed to be 22 points in both groups. Using 0.05 alpha, 0.9 force, and 1: 1 allocation, a sample size per group of 60 patients is obtained. The sample size calculation was performed using G * Power 3.1.5.1 (comparison of means, two-tailed, A priori). Approximately 10% of patients are estimated to disappear at follow-up, bringing the final sample size to a total of 133 patients.

Schedule, interim analysis and completion of the study

The study is planned to start in summer / autumn 2014. An interim analysis will be performed in the middle of the study, ie for 66 patients. If a statistically significant difference is obtained for the main response variable already at this stage, the study will not be continued. Otherwise, the study will continue until 133 patients have been randomized. It is estimated that it will take about five years to recruit patients. The follow-up phase will continue for another eight years.

Registration

The study is registered with clinicaltrials.gov before randomization begins.

Privacy Policy

Personal data of randomized patients are collected in the study folder, and CRF # 1, # 2, and # 3 are completed for them. Patients who meet the inclusion criteria and thus are evaluated for participation in the study will be accounted for, how many patients will be excluded from the study, and for what reason (fulfillment of the exclusion criterion, refusal, etc.). Personal data of patients who dropped out of the study will not be collected. The material to be collected is stored in a locked state and the electronic data is protected by passwords and encryption on computers.

References

Andersen JC, Bundgaard L, Elbrønd H, Laurberg S, Walker LR, Støvring J, Danish Surgical Society. 2012. Danish national guidelines for treatment of diverticular disease. Dan Med J 59:C4453.

Biondo S, Lopez Borao J, Millan M, Kreisler E, Jaurrieta E. 2012. Current status of the treatment of acute colonic diverticulitis: a systematic review. Colorectal Disease 14:e1–e11.

Chapman JR, Dozois EJ, Wolff BG, Gullerud RE, Larson DR. 2006. Diverticulitis: a progressive disease? Do multiple recurrences predict less favorable outcomes? Ann Surg 243:876–830; discussion 880–3.

Forgione A, Leroy J, Cahill RA, Bailey C, Simone M, Mutter D, Marescaux J. 2009. Prospective Evaluation of Functional Outcome After Laparoscopic Sigmoid Colectomy. Ann Surg 249:218–224.

Gaertner WB, Willis DJ, Madoff RD, Rothenberger DA, Kwaan MR, Belzer GE, Melton GB. 2013. Percutaneous Drainage of Colonic Diverticular Abscess. Dis Colon Rectum 56:622–626.

Pasternak I, Wiedemann N, Basilicata G, Melcher GA. 2011. Gastrointestinal quality of life after laparoscopic-assisted sigmoidectomy for diverticular disease. Int J Colorectal Dis 27:781–787.

Rafferty J, Shellito P, Hyman NH, Buie WD. 2006. Practice Parameters for Sigmoid Diverticulitis. Dis Colon Rectum 49:939–944.

Ritz J-P, MD KSL, MD BF, PhD AS, FRCS HJBMF, MD CH. 2011. Outcome of patients with acute sigmoid diverticulitis: Multivariate analysis of risk factors for free perforation. Surgery 149:606–613.

Wasvary H, Turfah F, Kadro O, Beauregard W. 1999. Same hospitalization resection for acute diverticulitis. Am Surg 65:632–5– discussion 636.
