# Supplementary material for: Sigmoid Resection vs Conservative Treatment After Diverticulitis: Prespecified 4-Year Analysis of the LASER Randomized Clinical Trial
Source: JAMA Surg. 2025 Apr 9;160(6):615–22. doi: 10.1001/jamasurg.2025.0572 (PMC11983291; doi:10.1001/jamasurg.2025.0572)
Supplement: Supplement 2. — eTable. Baseline Characteristic of Patients Included in Quality-of-Life Analyses [file jamasurg-e250572-s002.pdf]

Supplemental Online Content

Santos A, Mentula P, Pinta T, et al. Sigmoid resection vs conservative treatment after diverticulitis: prespecified 4-year analysis of the LASER randomized clinical trial. *JAMA Surg*. Published online April 9, 2025. doi:10.1001/jamasurg.2025.0572

eTable. Baseline Characteristic of Patients Included in Quality-of-Life Analyses

This supplemental material has been provided by the authors to give readers additional information about their work.

© 2025 Santos A et al. *JAMA Surgery*.

eTable. Baseline Characteristic of Patients Included in Quality-of-Life Analyses

	**Surgery (N = 38)**	**Conservative treatment (N = 40)**
**Age, median (IQR), y**	59 (52.8-63)	59 (51-63)
**Sex, male**	9 (24)	13 (33)
**Body Mass Index, mean (SD)**	29.5 (4.71)	28.7 (4.23)
**Comorbidities**		
Coronary disease/Myocardial infarction	0	1 (3)
Congestive heart failure	0	1 (3)
Atrial fibrillation	1 (3)	1 (3)
Hypertension	15(40)	11 (28)
Peripheral vascular disease	0	1 (3)
Cerebrovascular disease	0	0
Hemiplegia	0	0
Dementia	0	0
COPD or asthma	3 (8)	3 (8)
Connective tissue disease	2 (5)	2 (5)
Liver disease	0	0
Peptic ulcer	0	0
Diabetes mellitus	1 (3)	5 (13)
without complications	1 (3)	4 (10)
with complications	0	1 (3)
Kidney disease (moderate / severe)	0	0
Cancer	0	0
Leukemia	0	0
Lymphoma	0	0
AIDS	0	0
**Inclusion criteria#**		
Recurrent diverticulitis	31 (82)	29 (73)
Complicated diverticulitis##	10 (26)	11 (28)
Persistent pain >3 months after diverticulitis	2 (5)	3 (8)
**Frequency of pain at randomisation**		
never	3 (8)	3 (8)
once a month	11 (29)	6 (15)
once a week	7 (18)	7 (18)
few times a week	7 (18)	8 (20)
everyday	1 (3)	5 (13)
several times a day	0	1 (3)
all the time	2 (5)	1 (3)
**VAS score, mean (SD)**	4.2 (3.0)	4.8 (2.7)
**GIQLI at randomisation, mean (SD)**	103.41 (22.01)	100.44 (21.28)
**SF-36 at randomisation, median (IQR)**		
PCS	47.71 (42.31-53.58)	43.40 (36.70-50.82)
MCS	55.63 (44.65-60.16)	50.63 (41.88-57.76)
**Episodes of diverticulitis, mean (SD)**	4.7 (3.6)	3.9 (3.0)
**Most severe diverticulitis before randomisation**		
Hinchey 0	7 (18)	10 (25)
Hinchey Ia	22 (58)	18 (45)
Hinchey Ib	3 (8)	6 (15)
Hinchey II	3 (8)	5 (13)
Hinchey III	2 (5)	0

© 2025 Santos A et al. *JAMA Surgery*.

**Most invasive treatment for earlier diverticulitis**		
Symptomatic treatment	1 (3)	1 (3)
Antibiotics	27 (71)	26 (65)
**Most invasive treatment for earlier diverticulitis**		
Percutaneous drainage	6 (16)	8 (20)
Laparoscopic lavage	2 (5)	1 (3)
**Location of diverticulosis**		
whole colon	3 (8)	5 (11)
sigmoid	23 (61)	22 (55)
sigmoid and transverse	5 (12)	1 (2)
descending colon	7 (17)	12 (30)
**Earlier treatment of diverticulosis***		
No treatment	11 (29)	8 (20)
Repeated per oral antibiotics	21 (55)	23 (58)
Fibre supplement	21 (55)	23 (58)
Behavioural†	12 (32)	10 (25)
**Medication**		
Anticoagulative medication	3 (8)	1 (3)
Corticosteroid medication	0	0
Immunosuppressive medication	2 (5)	2 (5)

No significant differences were identified between the treatment groups in any baseline variables. Abbreviations : COPD – Chronic Obstructive Pulmonary Disease, AIDS – Acquired Immunodeficiency Syndrome, VAS – Visual Analog Scale, GIQLI – Gastrointestinal Quality of Life, SF-36 – Short Form 36, PCS – Physical component score, MCS – Mental component score

#One patient could met more than one inclusion criteria

##Complicated diverticulitis were abscess (10 in surgery group, 11 in conservative group),

*More than one form of treatment could be assigned per patient.

†Diet changes in order to accommodate higher levels of fibre intake. Treatment of constipation if necessary.

© 2025 Santos A et al. *JAMA Surgery*.
