# Supplementary material for: Sigmoid Resection vs Conservative Treatment After Diverticulitis: Prespecified 4-Year Analysis of the LASER Randomized Clinical Trial
Source: JAMA Surg. 2025 Apr 9;160(6):615–22. doi: 10.1001/jamasurg.2025.0572 (PMC11983291; doi:10.1001/jamasurg.2025.0572)
Supplement: Supplement 3. — Data Sharing Statement [file jamasurg-e250572-s003.pdf]

Data Sharing Statement

Santos. Sigmoid Resection vs Conservative Treatment After Diverticulitis. *JAMA Surg*.
Published April 09, 2025. doi:10.1001/jamasurg.2025.0572

Data
Additional Information: ClinicalTrials.gov (NCT02174926)
Data available: No

Additional Information
Explanation for why data not available: Study permissions do not allow data sharing.
